# Visual and Refractive Outcomes after Phacoemulsification Cataract Surgery in Nanophthalmic Eyes

**DOI:** 10.3390/jcm13195852

**Published:** 2024-09-30

**Authors:** Tracy H. T. Lai, Jeffrey Y. T. Tse, Jacqueline W. T. Chan, Kenneth K. W. Li

**Affiliations:** 1 Department of Ophthalmology, Tseung Kwan O Hospital, 2 Po Ning Lane, Tseung Kwan O, Hong Kong; 2 Department of Ophthalmology, United Christian Hospital, 130 Hip Wo Street, Kwun Tong, Hong Kong; 3 Department of Ophthalmology, School of Clinical Medicine, LKS Faculty of Medicine, The University of Hong Kong, 21 Sassoon Road, Pokfulam, Hong Kong; 4 Wu Ho Loo Ning Cataract Center, Tseung Kwan O Hospital, 2 Po Ning Lane, Tseung Kwan O, Hong Kong

**Keywords:** nanophthalmic eyes, phacoemulsification, cataract surgery, refractive outcomes

## Abstract

**Background/Objectives**: The aim of this study was to report the visual and refractive outcomes of nanophthalmic eyes undergoing phacoemulsification at a tertiary cataract center. **Methods:** This is a prospective consecutive case series. Patients with an axial length of ≤20.5 mm who underwent phacoemulsification at a tertiary cataract center in Hong Kong were included. Eyes undergoing extracapsular cataract extraction or with a previous history of intraocular surgery including trabeculectomy were excluded. The outcome measures were the corrected distance visual acuity (CDVA) and refractive status at four months post-operation. Different intraocular lens formulas were used to compare the refractive outcomes. **Results:** Out of 22,847 cataract surgeries performed from May 2011 to March 2020, 14 eyes (0.06%) of 10 patients had axial lengths of ≤20.5 mm and underwent phacoemulsification. The mean axial length was 20.13 ± 0.44 mm. Out of these fourteen eyes, three (21%) had postoperative myopic shift with spherical equivalent refraction of more than or equal to 1D compared to the original target. Eleven eyes (79%) had postoperative refraction within 0.5D compared to the original target. Nine out of fourteen eyes (64%) had improvements in postoperative vision. There were no intraoperative complications. When comparing the Hoffer Q, Holladay 1, Holladay 2, Haigis and Hill-RBF 2.0 formulas, there was no significant difference in the absolute errors between the five formulas (*p* = 0.072). **Conclusions:** There was no significant difference in the mean absolute errors between the Hoffer Q, Holladay 1, Holladay 2, Haigis and Hill-RBF 2.0 formulas. Myopic shift was not uncommon, and more studies on intraocular lens (IOL) power calculation for short eyes are warranted.

## 1. Introduction

Nanophthalmos is a rare anomaly characterized by short axial lengths ranging from less than 17 mm to 21 mm [1,2,3,4]. It represents a pure form of microphthalmos as there are no gross developmental defects found [5]. Nanophthalmos is characterized by a shallow anterior chamber, a narrow angle, high hyperopia, a small cornea and a thickened sclera [3,5,6].

Due to the shallow anterior chamber with a disproportionately large lens, 54–77% of patients with nanophthalmos develop angle-closure glaucoma [3,7,8]. Therefore, it is hypothesized that cataract extraction might relieve angle closure and achieve better control of intraocular pressure in nanophthalmos [2]. However, cataract surgery in nanophthalmos is technically challenging because of the narrow anterior chamber and reduced working space. It has been reported previously that such an operation is associated with a high risk of intraoperative and postoperative complications [9]. Common potential complications described include posterior capsular rupture, uveal effusion, aqueous misdirection and severe posterior uveitis [1,5,10,11,12,13,14,15]. Impaired vortex venous drainage via the thickened sclera results in choroidal congestion and subsequent uveal effusion, which may lead to serous retinal detachment requiring sclerectomy [5,16]. A higher risk of posterior capsular rupture is noted in nanophthalmic cataract surgery due to the small working space in the anterior chamber and high vitreous pressure. Furthermore, refractive outcomes after cataract surgery are highly unpredictable [14,17]. Intraocular lens power calculation is more challenging for nanophthalmos [10], and many eyes cannot achieve postoperative refraction within 1 diopter (D) of the target refraction [10,18]. According to a review published by Gupta et al., the prediction of effective lens position is the biggest source of error in IOL power measurement, accounting for 35.5% of inaccuracies, followed by axial length measurement (17%), keratometry measurement (10.1%) and postoperative refraction (27%) [19]. Short eyes requiring high IOL powers are especially affected by prediction errors of the effective IOL position.

Hoffer Q, a third-generation traditional formula, is commonly used in IOL biometry for short eyes. However, as it does not measure anatomic anterior chamber depth, it may be less reliable in eyes with abnormal anterior segments [19].

In addition to traditional formulas, many artificial intelligence-based formulas have been designed in recent years to try to improve IOL formula accuracy by leveraging large datasets. Examples of such formulas include “FullMonte”, “Ladas”, “Hill-RBF”, “PEARL-DGS”, “Kane”, “Karmona”, “Hoffer QST” and “Nallasamy” [20], and online calculators for new IOL formulas have become widely available [21]. In fact, a recent meta-analysis found artificial intelligence-based IOL formulas to be significantly more accurate than traditional IOL formulas in short eyes. A meta-analysis involving 1476 eyes by Luo found new-generation formulas such as Pearl-DGS and Okulix to be significantly more accurate than Barrett Universal II, Hoffer Q and Holladay II [22]. Another systematic review by Stopyra et al. found that PEARL-DGS achieved the highest percentage of patients with ±0.5 D in short eyes [23].

In this study, we describe the refractive outcomes and complications of phacoemulsification in nanophthalmic eyes (axial length ≤20.5 mm). The mean, median and root-mean-square absolute errors between different intraocular lens formulas were compared.

## 2. Materials and Methods

The data for this study were extracted from the database of the continuous prospective audit of all cataract surgeries performed at the Wu Ho Loo Ning Cataract Center, Tseung Kwan O Hospital, Kowloon East Cluster, Hong Kong, between May 2011 and March 2020. This center provides tertiary eye care to the eastern part of the Kowloon peninsula, with a population of 1.1 million. Nanophthalmos is defined as an axial length of ≤20.5 mm without morphological malformation. Eyes undergoing extracapsular cataract extraction or with a previous history of intraocular surgery including trabeculectomy were excluded. Indications for cataract surgery were visually significant cataract and acute angle-closure glaucoma after the stabilization of intraocular pressure. The minimum sample size, with a power of 0.8, a 95% confidence level and an effect size of 0.5, was 10–15. An IOLMaster 500 or 700 (Carl Zeiss Meditec AG, Jena, Germany) was used for biometry in all patients undergoing phacoemulsification. The IOLMaster 700 is a swept-source OCT biometer that operates at a wavelength of 1035 to 1080 nm to generate a 2D OCT cornea-to-retina cross-section scan of the eye in six meridians (0°, 30°, 60°, 90°, 120° and 150°). Each meridional scan is averaged from three single scans. This technology is used to derive all axial biometry measurements, including the axial lengths, central corneal thickness, anterior chamber depths from the epithelium and endothelium, and lens thickness [24]. For keratometry, the IOLMaster 700 measures the anterior corneal radius by using telecentric keratometry and simultaneously measures the posterior corneal radius and central corneal thickness (CCT) by using swept-source optical coherence tomography (SS-OCT). Its telecentric keratometry technology measures the anterior surface at 1.5, 2.5 and 3.2 mm diameters by using 18 projected reference points (equivalent to nine meridians). The posterior surface is mapped using six meridional SS-OCT scans [19]. In our series, all eyes were measurable using IOLMaster, and no eye required the use of an ultrasound A-scan for axial length measurement. The Hoffer Q formula was the standard formula used to determine the required IOL power in all cases of nanophthalmos. This study was conducted according to the tenets of the Declaration of Helsinki, and the study protocol was approved by the research ethics committee of the Kowloon East Cluster of the Hospital Authority of Hong Kong (Ref: KC/KE-20-0151/ER-3).

Table 1 shows the preoperative data, IOL formulae, power and type; Table 2 shows the patient demographics, preoperative and postoperative visual acuity, ocular comorbidity and IOP. Day 1 postoperative examination was performed in all cases. The patients were subsequently followed up at one week, one month and four months after surgery. Postoperative corrected distance visual acuity (CDVA) and spherical equivalent were recorded at four months after surgery. All intraoperative and postoperative complications and subsequent procedures were reviewed. Complications were defined as any issue requiring additional operations, except Neodymium:Yag (Nd:Yag) capsulotomy for posterior capsule opacification.

Surgical technique: A 2.2 mm or 2.75 mm clear corneal incision phacoemulsification was performed in all cases under local anesthesia. Local anesthesia was given by either topical anesthesia, retrobulbar injection or peribulbar block. Phacoemulsification was conducted through a temporal or superior approach. At the conclusion of surgery, intracameral cefuroxime was given routinely, except for cases with a known allergy to the cephalosporin or penicillin group of antibiotics [25]. No prophylactic sclerotomy was performed. Multiple surgeons with different levels of expertise performed the operations.

Data analysis: Preoperative and postoperative CDVA data, measured using Snellen charts, were converted to logMAR visual acuity for analysis. Microsoft Excel Version 2023 (Microsoft Corp., Redmond, WA, USA) and SPSS 29 (SPSS Inc., Chicago, IL, USA) were used for statistical analysis. Refraction was performed four months postoperatively by optometrists. The spherical equivalent, which is defined as the sphere power plus half the cylinder power, was taken as the refractive outcome. The prediction error of postoperative refraction was defined as the difference between the actual postoperative and the predicted postoperative spherical equivalent. The absolute error was calculated from the numerical value of each prediction error without regard to its sign. A Kruskal–Wallis test was used to check for any differences in the absolute error among the Hoffer Q, Holladay 1, Holladay 2, Haigis and Hill-RBF 2.0 formulas. A Kruskal–Wallis test was used as the data were not normally distributed and the sample size was small (*n* = 14 for each group). The difference between the actual and the predicted postoperative refraction was calculated using the formula Prediction error = Actual postoperative spherical equivalent—Predicted spherical equivalent. The mean, median and root-mean-square absolute errors were also reported. The root-mean-square absolute error is an alternative to the standard deviation for describing the distribution of prediction errors between different groups.

## 3. Results

### 3.1. Patient Characteristics

**Figure 1:**
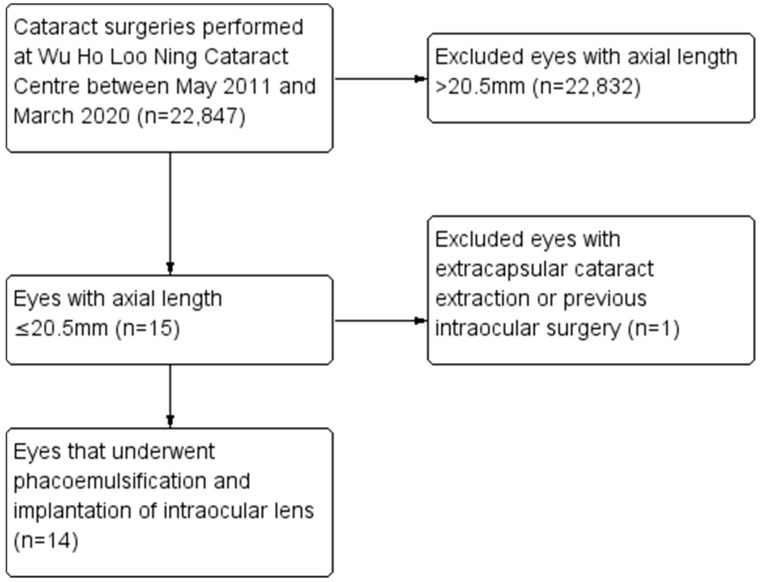
Flowchart of included population.

During the study period, 22,847 cataract surgeries were performed. Out of these, a total of 14 nanophthalmic eyes (0.06%) of 10 patients who underwent phacoemulsification were identified. All 10 patients were of Asian descent. Figure 1 shows a flowchart of case selection. A total of 14 eyes of 10 patients (9 females, 1 male) with a mean age of 72 (range 63 to 83) were included. The mean axial length was 20.13 ± 0.44 mm (range 18.82 to 20.49 mm). The mean anterior chamber depth was 2.40 ± 0.36 mm (range 1.97 to 3.02 mm). Visually significant comorbidities were present in seven eyes (50%). These included diabetic maculopathy (two eyes), age-related macular degeneration (two eyes), a history of acute angle-closure glaucoma (one eye) and corneal scar (two eyes). Previous laser peripheral iridotomy was performed in six eyes (43%). No eyes had received previous trabeculectomy or vitrectomy. Table 2 shows the demographic and preoperative data of the study population.

### 3.2. Surgery

Single-piece acrylic IOLs were successfully implanted in the capsular bag for all eyes. The IOL power ranged from +27D to +35D. No complications, such as posterior capsule rupture, Descemet membrane detachment and choroidal hemorrhage, were reported. There was one case with mild iris prolapse from the main wound that occurred intraoperatively, which was repositioned using viscoelastics.

### 3.3. Visual and Refractive Outcomes

The mean preoperative logMAR visual acuity was 0.63 ± 0.25 (Snellen visual acuity of 20/80), while the mean logMAR CDVA at 4 months after surgery was 0.37 ± 0.19 (Snellen visual acuity of 20/50). The Snellen visual acuity of hand movement was taken to be equivalent to a logMAR visual acuity of 2.28 [26]. Nine eyes (64%) showed improvement in visual acuity and five eyes (36%) did not have improvement in visual acuity. The reasons were pre-existing ocular co-morbidities such as diabetic maculopathy, age-related macular degeneration and corneal scar. None of the cases had loss of visual acuity.

Using the Hoffer Q formula, 11 eyes (79%) achieved a final spherical equivalent within ±1D of the target refraction (Table 3). Three out of fourteen eyes (21%) had postoperative myopic shift with a spherical equivalent refraction of ≥1D compared to the original target. Table 4 shows the difference between the actual and predicted target spherical equivalents. The mean predicted error was −0.46D for Hoffer Q. The predicted errors of the Holladay 1, Holladay 2, Haigis and Hill-RBF 2.0 formulas were also calculated by subtracting the predicted refraction based on the IOL actually implanted in the eye from the actual postoperative refraction. The mean predicted errors were −0.46D for Hoffer Q, −0.24D for Holladay 1, −0.18D for Holladay 2, 0.60D for Haigis and 0.22D for Hill-RBF 2.0. The mean absolute errors were also estimated from the numerical value of each prediction error without regard to its sign. The mean absolute error was 0.55D for Hoffer Q, 0.58D for Holladay 1, 0.60D for Holladay 2, 0.90D for Haigis and 0.89D for Hill-RBF 2.0. Table 4 compares the predicted errors using the Hoffer Q, Holladay 1, Holladay 2, Haigis and Hill-RBF 2.0 formulas. For one eye, only the Hoffer Q formula could be used as it was out of range for Holladay 1, Holladay 2, Haigis and Hill-RBF 2.0. The differences in absolute errors between the five formulas were assessed using the Kruskal–Wallis test. Although the Hoffer Q formula had the smallest mean absolute error and root-mean-square absolute error, while Holladay 1 yielded the smallest median absolute error, there was no statistically significant difference in the absolute errors between the five formulas (*p* = 0.072).

## 4. Discussion

This study evaluates the visual outcomes, refractive outcomes and intraoperative complications of nanophthalmic cataract surgery.

In the present study, 64% of eyes showed improvements in visual acuity postoperatively, and no eyes lost vision. No intraoperative or postoperative complications were reported. Four eyes (31%) gained ≥3 Snellen lines, which was comparable to another study by Steijns et al. which showed an improvement of ≥3 Snellen lines in 19 out of 43 eyes (44%) [10]. 

Accurate intraocular lens power calculation is crucial in cataract surgery in order to avoid unnecessary refractive surprises. Refractive surprises are more common for eyes with nanophthalmos [10]. Shallow anterior chamber depth, short axial length, small corneal diameter, high-power IOL and high posterior capsular rupture rates are the various factors contributing to poor refractive outcomes [12]. Short eyes requiring high-power IOL are more significantly affected by inaccuracies in predicting postoperative IOL position [19]. Moreover, IOLs over 30 D are only required to be within ±1.00 D of the labeled power compared with ±0.50 D for IOLs less than 30D [27].

Numerous studies have been conducted to compare the accuracy of various traditional and artificial intelligence-based new IOL formulas. There is controversy regarding the most accurate IOL formula in short eyes [9].

Similar to the result of our study, two studies suggested that the Hoffer Q formula may be more accurate for short eyes. A case series of 100 short eyes by Vilaltella et al. found that Hoffer Q obtained the lowest median absolute error out of 10 traditional and artificial intelligence-based IOL formulas [28], although statistically not significant. Another series of 62 eyes by Stopyra found that Hoffer Q yielded the lowest mean absolute error out of six IOL formulas [29].

The Kane formula was found to be the most accurate in two large-scale studies involving 766 [30] and 625 [31] short eyes, outperforming formulas such as Holladay 1, Hill-RBF, Hoffer Q, Haigis, SRK/T and Barrett-Universal II [32].

Meta-analyses comparing different IOL formulas were conducted. A meta-analysis of 1476 short eyes by Luo et al. found that Pearl-DGS had the highest percentage of eyes within ±0.5D, significantly higher than Barrett Universal II, Haigis, Hoffer Q, Holladay1, Holladay2 and Olsen [22]. Another systematic review of more than 3000 short eyes by Stopyra et al. found that Barrett achieved the smallest mean absolute error and PEARL-DGS achieved the highest percentage of patients with ±0.5 D in short eyes [23].

While most studies looked into the refractive outcomes of short eyes with an axial length of <22 mm, our study focused on nanophthalmic eyes with an axial length of ≤20.5 mm. We found no statistically significant difference in the mean absolute error among the Hoffer Q, Holladay 1, Holladay 2, Haigis and Hill-RBF 2.0 formulas (*p* = 0.072). In our series, 11 eyes (79%) achieved a final refraction within ±1D of the target refraction. Three eyes (21%) achieved a final refraction ≥1D lower than the target refraction. We observed a tendency toward myopic shift. In daily clinical practice, we could consider aiming for plano instead of −0.5D when choosing IOLs for nanophthalmic eyes to reduce the risk of refractive surprise due to myopic shift.

Although nanophthalmos is primarily defined by a short axial length, the exact cutoff for axial length vary among studies, rendering comparisons of surgical outcomes and complications among different studies complicated. As we only included eyes with an axial length of ≤20.5 mm, eyes with relative anterior nanophthalmos [33] were not studied. These eyes have a normal axial length but a shallow anterior chamber depth, which may increase the risk of refractive surprise. This study is mainly limited by the small sample size, a common problem shared by previously published case series. A prospective multicenter study is needed to provide more clinical evidence and data on this uncommon but important clinical entity. In addition, the cataract surgeries were not performed by a single surgeon. There is variability in the level of surgical expertise. The surgically induced astigmatism (SIA) of each surgeon may be different due to different techniques of main wound construction. The variability in wound size (2.2 or 2.75 mm) may also affect the refractive outcome.

## 5. Conclusions

In conclusion, this study confirms that nanophthalmos is exceedingly rare in our local population receiving cataract surgery, which is 0.06% in our series. Among the Hoffer Q, Holladay 1, Holladay 2, Haigis and Hill-RBF 2.0 formulas, we report that Hoffer Q has the smallest mean absolute error and root-mean-square absolute error, although the difference is not statistically significant. We also observe myopic shift of ≥1D in 21% of cases. In daily clinical practice, we could consider aiming for plano instead of −0.5D when choosing IOLs for nanophthalmic eyes to reduce the risk of refractive surprise due to myopic shift. A multicenter study with a larger sample size of nanophthalmic eyes to identify the IOL formula with the smallest absolute error is warranted. Additional artificial intelligence-based formulas could also be included in future studies.

Cataract surgery in nanophthalmos remains challenging and is associated with significant risks of complications and refractive surprise. Careful preoperative assessment, counseling and appropriate precautions during surgery are important in these high-risk patients. 

## Figures and Tables

**Table 1:** Preoperative data, IOL formula, power and type.

Patient	Eye	Age	Sex	Axial Length (mm)	Preoperative ACD (mm)	K1	K2	Formula	IOL Power (D)	IOL Type
1	L	63	F	20.36	2.84	48.25	48.87	Hoffer Q	27.5	HOYA PY60AD
	R	64		20.49	2.95	48.87	49.25	Hoffer Q	27	Alcon SN60WF
2	L	72	F	18.82	2.44	46.50	48	Hoffer Q	35	Alcon SN60WF
	R	74		20.48	2.3	46.75	47.75	Hoffer Q	29	AMO ZCB00
3	L	73	F	20.13	2.23	47.67	49.78	Hoffer Q	30	AMO ZCB00
4	R	83	M	20.14	2.15	44.00	47.47	Hoffer Q	33	AMO ZCB00
5	R	73	F	19.83	3.02	46.94	48.21	Hoffer Q	33.5	AMO ZCB00
	L	75		19.82	2.62	46.94	47.47	Hoffer Q	33.5	AMO ZCB00
6	R	76	F	19.91	1.97	47.07	47.87	Hoffer Q	33	AMO ZCB00
7	R	68	F	20.45	2.09	50.90	51.29	Hoffer Q	25.5	Alcon SN60WF
	L	71		20.26	2.02	49.63	51.29	Hoffer Q	27	Alcon SN60WF
8	R	79	F	20.46	2.09	45.00	46.55	Hoffer Q	32.5	AMO ZCB00
9	R	64	F	20.2	2.47	48.21	48.63	Hoffer Q	30	Alcon SN60WF
10	L	73	F	20.41	2.65	43.77	44.18	Hoffer Q	34	AMO ZCB00

ACD = anterior chamber depth; mm = millimeter; L = left; R = right; K1 = flat keratometry; K2 = steep keratometry; IOL = intraocular lens; D = diopter.

**Table 2:** Demographics and preoperative data of the study population.

Sex (Male/Female)	1 Male, 9 Female
Age (years)	
Mean ± SD	72 ± 5.33
Range	63–83
Preoperative visual acuity (logMAR)	
Mean ± SD	0.63 ± 0.25
Postoperative visual acuity (logMAR)	
Mean ± SD	0.37 ± 0.19
Axial length (mm)	
Mean ± SD	20.13 ± 0.44
Range	18.82–20.49
Anterior chamber depth (mm)	
Mean ± SD	2.40 ± 0.36
Range	1.97–3.02
IOL power (D)	
Mean	30.5
Range	25.5–35
Cataract grading	
≥III:	4
I–II:	10

SD = standard deviation; D = diopter.

**Table 3:** Difference between the actual and predicted spherical equivalent at 4 months after surgery.

Difference between Postoperative Refraction and Target Refraction	No. of Eyes	%
Postoperative refraction of ≥2.5D lower than target refraction	0	0
Postoperative refraction of 1.0–2.5D lower than target refraction	3	21
Postoperative refraction within ±1.0D of target refraction	11	79
Postoperative refraction of 1.0–2.5D higher than target refraction	0	0
Postoperative refraction of ≥2.5D higher than target refraction	0	0
Total	14	100

D = diopter.

**Table 4:** Predicted SE and Actual SE. Predicted errors, mean and median absolute errors, and root-mean-square absolute errors for Hoffer Q, Holladay 1, Holladay 2, Haigis and Hill-RBF 2.0 formulas.

Patient	Eye	Predicted SE (Hoffer-Q)	Actual SE	Predicted Error (Hoffer-Q)	Predicted Error (Holladay 1)	Predicted Error (Holladay 2)	Predicted Error (Haigis)	Predicted Error (Hill-RBF 2.0)
1	L	−0.18	−0.5	−0.32	0.21	−0.26	0.44	0.22
	R	−0.69	−0.5	0.19	0.16	−0.41	0.46	0.47
2	L	0.09	−0.25	−0.34	Not in range	Not in range	Not in range	Not in range
	R	−0.53	−2.625	−2.1	−2.65	−2.535	−1.955	−2.02
3	L	−0.6	−1.625	−1.03	−0.78	−0.715	0.135	0.02
4	R	0.2	−0.25	−0.45	0.14	0.44	0.79	0.7
5	R	−0.6	−1.375	−0.78	−0.11	−0.785	0.425	0.94
	L	−0.3	−0.5	−0.2	0.43	0.14	1.12	1.3
6	R	−0.5	−0.875	−0.38	0.23	0.495	1.305	1.17
7	R	−0.7	−0.25	0.45	−1.68	1.38	1.94	−2.1
	L	−0.5	−1.5	−1	−0.16	−0.19	0.55	−0.23
8	R	−0.7	−0.75	−0.05	0.39	−0.16	1.11	0.93
9	R	−0.6	−1	−0.4	0.19	0.02	0.82	0.65
10	L	−0.1	−0.125	−0.03	0.465	0.265	0.605	0.84
Mean predicted error				−0.46	−0.24	−0.18	0.60	0.22
Mean absolute error				0.55	0.58	0.60	0.90	0.89
Median absolute error				0.39	0.23	0.41	0.79	0.84
Root-mean-square absolute error				0.76	0.93	0.89	1.05	1.08

L = left; R = right; SE = spherical equivalent.

## Data Availability

The original contributions presented in this study are included in the article. Further inquiries can be directed to the corresponding author/s.

## References

[1] Yuzbasioglu E., Artunay O., Agachan A., Bilen H. (2009). Phacoemulsification in patients with nanophthalmos. Can. J. Ophthalmol. J. Can. D’Ophtalmol..

[2] Yalvac I.S., Satana B., Ozkan G., Eksioglu U., Duman S. (2008). Management of glaucoma in patients with nanophthalmos. Eye.

[3] Singh O.S., Simmons R.J., Brockhurst R.J., Trempe C.L. (1982). Nanophthalmos: A perspective on identification and therapy. Ophthalmology.

[4] Tay T., Smith J.E., Berman Y., Adès L., Missotte I., Saglibène H., Martin F., Mitchell P., Taylor D. (2007). Nanophthalmos in a Melanesian population. Clin. Exp. Ophthalmol..

[5] Brockhurst R.J. (1974). Nanophthalmos with uveal effusion: A new clinical entity. Trans. Am. Ophthalmol. Soc..

[6] Susanna R. (1987). Implantation of an intraocular lens in a case of nanophthalmos. Eye Contact Lens.

[7] Ritch R., Chang B.M., Liebmann J.M. (2003). Angle closure in younger patients. Ophthalmology.

[8] Wladis E.J., Gewirtz M.B., Guo S. (2006). Cataract surgery in the small adult eye. Surv. Ophthalmol..

[9] Elhusseiny A.M., Sallam A.B. (2023). Cataract surgery in adult eyes with short axial length. Curr. Opin. Ophthalmol..

[10] Steijns D., Bijlsma W.R., Van der Lelij A. (2013). Cataract surgery in patients with nanophthalmos. Ophthalmology.

[11] Day A.C., MacLaren R.E., Bunce C., Stevens J.D., Foster P.J. (2013). Outcomes of phacoemulsification and intraocular lens implantation in microphthalmos and nanophthalmos. J. Cataract Refract. Surg..

[12] Jung K.I., Yang J.W., Lee Y.C., Kim S.Y. (2012). Cataract surgery in eyes with nanophthalmos and relative anterior microphthalmos. Am. J. Ophthalmol..

[13] Sharan S., Grigg J.R., Higgins R.A. (2006). Nanophthalmos: Ultrasound biomicroscopy and Pentacam assessment of angle structures before and after cataract surgery. J. Cataract Refract. Surg..

[14] Wu W., Dawson D.G., Sugar A., Elner S.G., Meyer K.A., McKey J.B., Moroi S.E. (2004). Cataract surgery in patients with nanophthalmos: Results and complications. J. Cataract Refract. Surg..

[15] Faucher A., Hasanee K., Rootman D.S. (2002). Phacoemulsification and intraocular lens implantation in nanophthalmic eyes: Report of a medium-size series. J. Cataract Refract. Surg..

[16] Jackson T.L., Hussain A., Salisbury J., Sherwood R., Sullivan P.M., Marshall J. (2012). Transscleral albumin diffusion and suprachoroidal albumin concentration in uveal effusion syndrome. Retina.

[17] Oshika T., Imamura A., Amano S., Eguchi S., Nakayama M., Emi K. (2001). Piggyback foldable intraocular lens implantation in patients with microphthalmos. J. Cataract Refract. Surg..

[18] Inatomi M., Ishii K., Koide R., Kora Y., Ozawa T. (1997). Intraocular lens power calculation for microphthalmos. J. Cataract Refract. Surg..

[19] Gupta V., Pal H., Sawhney S., Aggarwal A., Vanathi M., Luthra G. (2024). Optimization of biometry for best refractive outcome in cataract surgery. Indian J. Ophthalmol..

[20] Stopyra W., Cooke D.L., Grzybowski A. (2024). A Review of Intraocular Lens Power Calculation Formulas Based on Artificial Intelligence. J. Clin. Med..

[21] Buonsanti D., Raimundo M., Findl O. (2024). Online intraocular lens calculation. Curr. Opin. Ophthalmol..

[22] Luo Y., Li H., Gao L., Du J., Chen W., Gao Y., Ye Z., Li Z. (2022). Comparing the accuracy of new intraocular lens power calculation formulae in short eyes after cataract surgery: A systematic review and meta-analysis. Int. Ophthalmol..

[23] Stopyra W., Langenbucher A., Grzybowski A. (2023). Intraocular Lens Power Calculation Formulas—A Systematic Review. Ophthalmol. Ther..

[24] Klaproth O., Aramberri J., Hoffer K.J., Olsen T., Savini G., Shammas H.J. (2024). ZEISS IOLMaster 700. Intraocular Lens Calculations.

[25] Ng A.L., Tang W.W., Li P.S., Li K.K. (2016). Intracameral cefuroxime in the prevention of postoperative endophthalmitis: An experience from Hong Kong. Graefe’s Arch. Clin. Exp. Ophthalmol..

[26] Lange C., Feltgen N., Junker B., Schulze-Bonsel K., Bach M. (2009). Resolving the clinical acuity categories “hand motion” and “counting fingers” using the Freiburg Visual Acuity Test (FrACT). Graefe’s Arch. Clin. Exp. Ophthalmol..

[27] Hoffer K.J., Savini G. (2017). IOL Power Calculation in Short and Long Eyes. Asia-Pac. J. Ophthalmol..

[28] Vilaltella M., Cid-Bertomeu P., Huerva V. (2023). Accuracy of 10 IOL power calculation formulas in 100 short eyes (≤22 mm). Int. Ophthalmol..

[29] Stopyra W. (2022). Effectiveness, Sensitivity, and Specificity of Intraocular Lens Power Calculation Formulas for Short Eyes. Turk. J. Ophthalmol..

[30] Darcy K., Gunn D., Tavassoli S., Sparrow J., Kane J.X. (2020). Assessment of the accuracy of new and updated intraocular lens power calculation formulas in 10 930 eyes from the UK National Health Service. J. Cataract Refract. Surg..

[31] Melles R.B., Holladay J.T., Chang W.J. (2018). Accuracy of Intraocular Lens Calculation Formulas. Ophthalmology.

[32] Kane J.X., Chang D.F. (2021). Intraocular Lens Power Formulas, Biometry, and Intraoperative Aberrometry: A Review. Ophthalmology.

[33] Hoffman R.S., Vasavada A.R., Allen Q.B., Snyder M.E., Devgan U., Braga-Mele R. (2015). Cataract surgery in the small eye. J. Cataract Refract. Surg..

